# Efficacy of ethyl-4-bromophenyl carbamate on different *Rhipicephalus microplus* stages implanted in cattle

**DOI:** 10.1007/s10493-023-00846-8

**Published:** 2023-10-03

**Authors:** Sandra Lizeth Iturbe-Requena, César Cuenca-Verde, María Guadalupe Prado-Ochoa, Víctor Hugo Vázquez-Valadez, Marco Antonio Muñoz-Guzmán, Enrique Angeles, Fernando Alba-Hurtado

**Affiliations:** 1 Departamento de Ciencias Biológicas, Facultad de Estudios Superiores Cuautitlán, Universidad Nacional Autónoma de México, Mexico City, Mexico; 2 Laboratorio de Química Medicinal, Departamento de Ciencias Químicas, Facultad de Estudios Superiores Cuautitlán, Universidad Nacional Autónoma de México, Mexico City, Mexico

**Keywords:** Acaricides, Carbamates, Efficacy, Pen test, Cattle ticks

## Abstract

The effect of ethyl-4-bromophenyl carbamate on different *Rhipicephalus microplus* stages implanted in cattle was evaluated using the pen test with infestation chambers. Twelve steers were distributed into four groups (n = 3), each with four chambers (12 chambers per group), where approximately 1,000 *R. microplus* larvae were placed in each chamber. The chambers of the first group were sprayed with a solution of ethyl-4-bromophenyl carbamate (0.668 mg/mL) on day 2 post-infestation (PI) (exposed larvae). The chambers of the second group were sprayed with the same solution on day 8 PI (exposed nymphs), and the chambers of the third group were sprayed on day 16 PI (exposed adults) with the same solution. The chambers of the fourth group were used as controls. The percentages of engorged females, egg laying, egg production and egg hatching were evaluated in all groups. The percentage of cumulative reduction of hatched larvae was 98.3, 96.1 and 94.4% when larvae, nymph and adult stages were treated, respectively. The average cumulative reduction of hatched larvae, considering the three treated stages, was 96.3%, whereby the reproductive potential of this tick was drastically reduced. In conclusion, ethyl-4-bromophenyl carbamate acted as an ixodicide (lethal effect) when larval stages were sprayed and as a growth regulator when nymphal and adult stages were sprayed. The sum of these effects had a direct impact on the efficacy of the product in the pen test, and future studies will indicate the potential use of this product for tick control.

## Introduction

Ticks are the most important hematophagous ectoparasites of cattle in tropical and subtropical areas of Latin America and other regions of the world (Andreotti et al. 2021), and infestation causes severe economic losses in cattle production. *Rhipicephalus microplus* and *Rhipicephalus annulatus* are the most important cattle ticks worldwide, and are the major vectors of *Babesia*, *Anaplasma*, and other rickettsiae (Domingos et al. 2013; Abbas et al. 2014). The use of chemical acaricides is the most common method of tick control. The regular and rational use of commercial acaricides produces selective pressure on tick populations for the selection of long-term resistant strains; however, their indiscriminate use accelerates this process (Guerrero et al. 2012). This negative result has generated the need for strategic use of the chemicals available on the market to increase their usefulness (George et al. 2004), as well as to design, synthesize, and test new molecules for tick control (Alba-Hurtado & Muñoz-Guzmán, 2021).

The ethyl-4-bromophenyl carbamate was designed and synthesized at the National Autonomous University of Mexico (Angeles et al. 2000). The effect of this compound on *R. microplus*, both susceptible and resistant to conventional ixodicide strains, has been evaluated in vitro, where it was observed to inhibit egg laying by more than 60% and egg hatching by up to 100% (Prado-Ochoa et al. 2013; Pérez-González et al. 2014). The observed biological effects of this ethyl-carbamate is the alteration of vitellogenesis and oocyte development, embryogenesis inhibition, and apoptosis induction in different cells of *R. microplus*, which occurs independently of acetylcholinesterase inhibition (Prado-Ochoa et al. 2014c; Iturbe-Requena et al. 2020b; Escobar-Chavarría et al. 2021). This ethyl-carbamate has low acute and subchronic toxicity in mammals, low genotoxic and mutagenic potential, and low ecotoxicity (Prado-Ochoa et al. 2014a, b, 2016; Iturbe-Requena et al. 2019, 2020a). Therefore, the objective of this study was to evaluate the effect of 4-ethyl-bromophenyl carbamates on the viability and some reproductive parameters of *R. microplus* implanted on the skin of bovines.

The results obtained from the in vitro tests in the laboratory showed the activity of this ethyl-carbamate on different developmental stages of *R. microplus*, which suggests that it could significantly inhibit the development of ticks implanted in cattle.

## Materials and methods

### Ethyl-carbamate

**Fig. 1:**
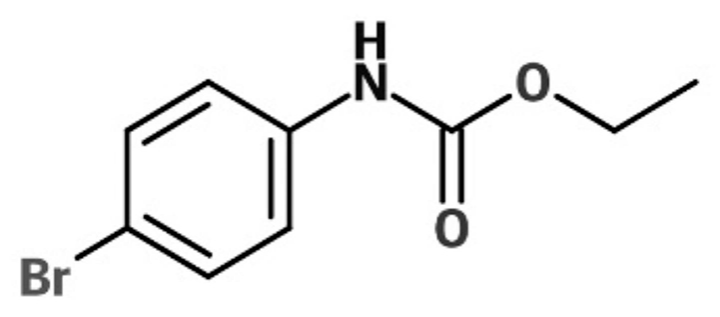
Chemical structure of ethyl-4-bromophenyl carbamate

The ethyl-4-bromophenyl carbamate was designed and synthesized at the Facultad de Estudios Superiores Cuautitlán of the National Autonomous University of Mexico (Angeles et al. 2000). This ethyl-carbamate was synthesized using halogenated derivatives of aromatic amines with ethyl-chloroformate using sodium bicarbonate and acetone as the reaction medium, and was subsequently purified by column chromatography and recrystallized. The structure was elucidated through spectroscopic techniques. The chemical structure of this compound is shown in Fig. 1, its molecular weight is 244 g/mol.

### Tick strain

*Rhipicephalus microplus* ticks from the ‘San Alfonso’ strain were used, which is resistant to organophosphates, pyrethroids, and amidines. The parasitic stages were maintained through controlled infestations in cattle located in a farm warehouse at, on average, 25 °C and 60–70% relative humidity (RH), and the nonparasitic stages were maintained in vitro in the laboratory in an incubator at 28 ± 2 °C with 80–90% RH. The strain was originally donated by the National Animal Health Verification Services Center of Mexico (Jiutepec, Morelos, Mexico). This strain has been maintained at the Facultad de Estudios Superiores Cuautitlán of the National Autonomous University of Mexico (9°38′38.0″N, 99°12′57.5″W; 2280 m above sea level) by our group and used in previous studies (Pérez-González et al., 2014; Iturbe-Requena et al. 2020b; Escobar-Chavarría et al. 2021).

### Animals

Twelve clinically healthy Holstein-Fresian steers (100–150 kg) were used, maintained from birth in tick-free conditions. The steers were housed in individual metal pens with slat floor (1 m wide × 2.2 m long × 1.2 m high) whose design partially limits movement to prevent the animal from detaching the chambers with ticks from its body through natural behavior. The steers feed consisted of dry alfalfa and water *ad libitum*. The maintenance portions were calculated based on the weight and age of the animals. This study was conducted according to the ARRIVE guidelines v.2.0 (Percie du Sert et al. 2020) and approved by the Internal Committee for Care of Experimental Animals of the Facultad de Estudios Superiores Cuautitlán, UNAM, Mexico (protocol no. C1305).

### Infestation of bovines

**Fig. 2:**
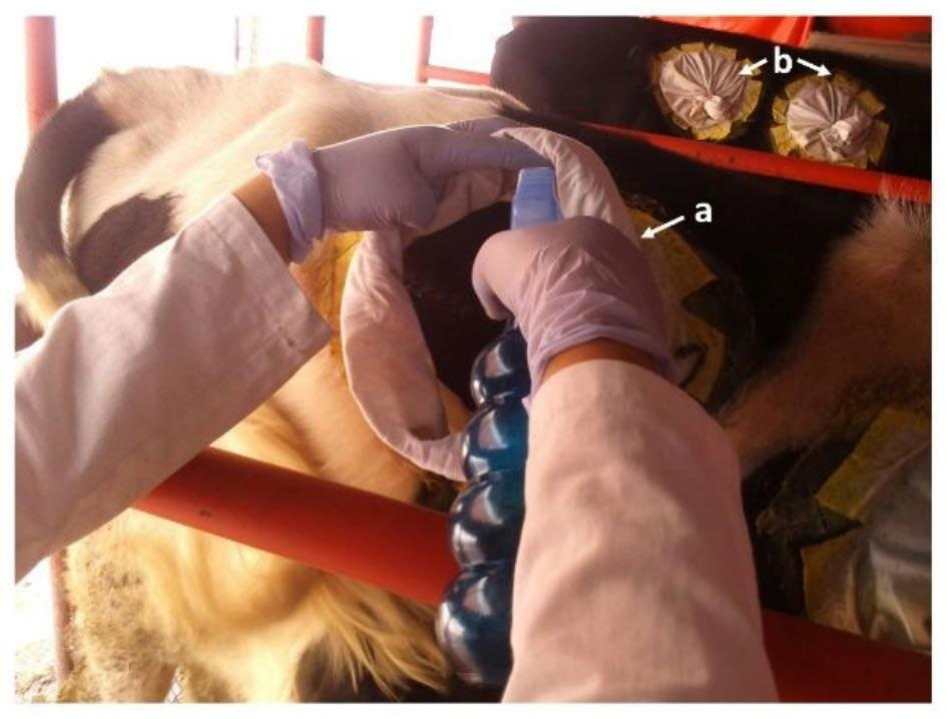
The pen test with infestation chambers. **(a)** Spraying with ethyl-4-bromophenyl carbamate solution to the chamber containing *Rhipicephalus microplus* ticks. **(b)** Closed chambers on steer

The evaluation of the ethyl-4-bromophenyl carbamate effect on *R. microplus* stages was carried out using the chamber test, which is a pen test proposed by Downing et al. (1977), and is described in the official Mexican standard NOM-006-ZOO-1993. On the upper region of both flanks of the steers, only the perimeter of four circles (two per side) of 20 cm diameter was shaved. In each circle a cotton fabric chamber (20 cm diameter, 30 cm long) was glued with a contact adhesive (Resistol 5000®), inside of which ticks were housed (Fig. 2).

### Experimental design

The steers were randomly distributed into four groups (n = 3). Four chambers (12 chambers per group) were placed on each steer, and approximately 1,000 tick larvae (total of larvae hatched from 60 mg of eggs) were infested in each chamber. The ethyl-carbamate was dissolved in dimethyl sulfoxide, and this solution was dissolved in distilled water. The final concentration of ethyl-carbamate was 0.668 mg/mL, and that of dimethyl sulfoxide was 4%. This was the 99% inhibitory concentration of egg hatching previously obtained in vitro (Pérez-González et al. 2014).

The chambers of the first group of steers were sprayed on day 2 post-infestation (PI) with 200 mL of the ethyl-carbamate solution (exposed larvae). The chambers of the second group of steers were sprayed on day 8 PI (exposed nymphs), and the chambers of the third group of steers were sprayed on day 16 PI (exposed adults) with the same ethyl-carbamate solution. The chambers of the fourth group of steers were sprayed with 200 mL of 4% dimethyl sulfoxide solution (ethyl-carbamate-free) and were used as a control group. Subsequently, in triplicate, 10 engorged females collected from each chamber were weighed, attached dorsally with double-sided masking tape inside Petri dishes, and incubated for 15 days at 28 ± 2 °C with 80–90% RH. After incubation from each Petri dish, the females laying eggs were counted, the egg mass was weighed and incubated for 21 days at 28 ± 2 °C with 80–90% RH, and the larvae hatched were counted. With the above data, the following parameters were calculated: % females laying eggs (= 100% × no. females laying eggs / total no. females), % eggs produced (= 100% × [weight of eggs laid / weight of females]_treated group_ / [weight of eggs laid / weight of females]_control group_), and % egg hatching (= 100% × no. egg shells / [no. egg shells + no. non-hatched eggs]) (Prado-Ochoa et al. 2013).

The % cumulative reduction of hatched larvae (i.e., the decrease of larvae produced for the next generation) was calculated as [1−(*abcd*)] × 100%, where *a* is the relative frequency of engorged females (= % engorged females/100), *b* is the relative frequency of egg laying (= % egg laying/100), *c* is the relative frequency of egg production (= % eggs produced/100), and d is the relative frequency of egg hatching (= % egg hatching/100).

### Statistical analysis

Percentages of engorged females, females laying eggs, egg produced, and egg hatching were analyzed with Graph Pad Prism statistical software by one-way ANOVA, followed by multiple comparison of means with Tukey’s test with a confidence level of 95%.

## Results

**Fig. 3:**
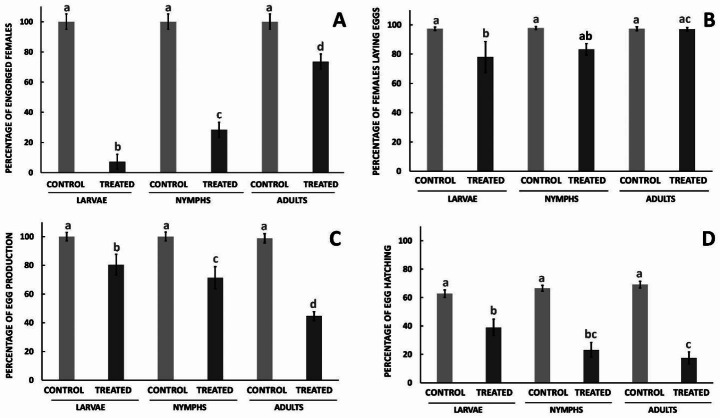
Effect of spraying ethyl-4-bromophenyl carbamate on three *Rhipicephalus microplus* stages in the pen test with infestation chambers: mean (± SE) percentage of **(A)** engorged females, **(B)** egg-laying females, **(C)** egg production, and **(D)** egg hatching. Means within a panel capped with different letters are significantly different (Tukey’s test: p < 0.05)

**Fig. 4:**
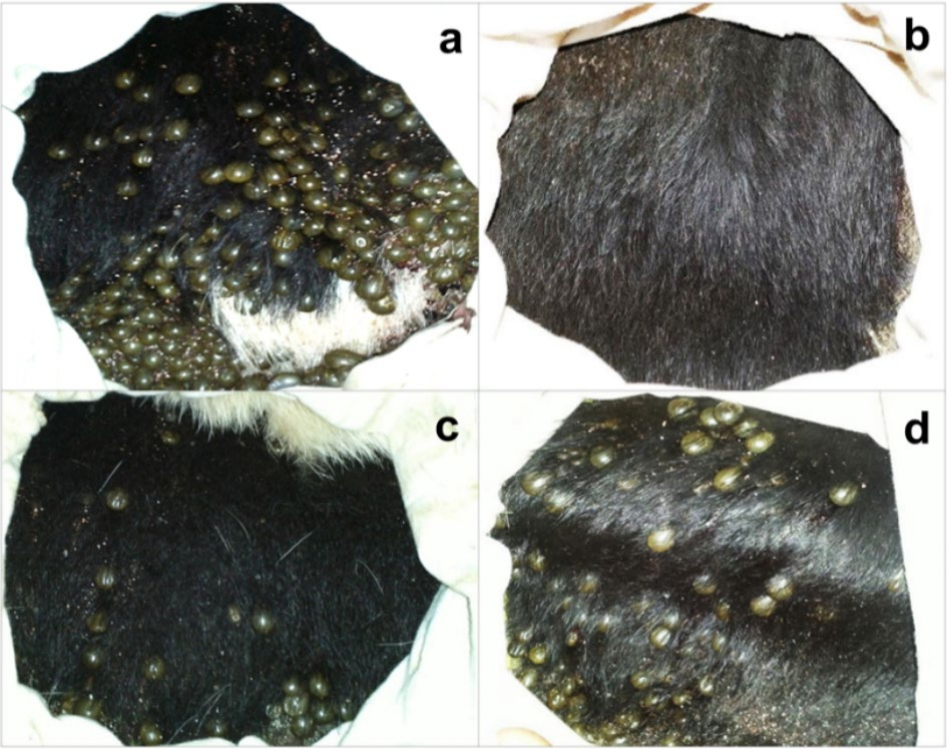
The pen test with infestation chambers showing *Rhipicephalus microplus* engorged females inside the chambers on day 21 post-infestation. Treatment with ethyl-4-bromophenyl carbamate was applied at various stages: **(a)** control (treated with dimethyl sulfoxide), and treated at the **(b)** larval, **(c)** nymphal, or **(d)** adult stage

A group effect (F_5,57_ = 75.85, p < 0.0001) was observed on the % engorged females. Analyzed by Tukey´s test, the % engorged females was lower when larvae were treated with ethyl-carbamate than when nymphs (p < 0.03) and adults (p < 0.0001) were treated (Fig. 3A). The treated nymphs resulted in a lower % engorged females (p < 0.0001) than the treated adults. Figure 4 shows representative photographs of the interior of the chambers with engorged females observed in the various treatment groups.

A group effect (F_5,124_ = 4.95, p < 0.001) was observed on the % females laying eggs. The % females laying eggs was lower (Tukey’s test: p < 0.05) when larvae were treated with ethyl-carbamate, compared with those in the control group. When nymphs and adults were treated, no differences were observed (p > 0.05) with their respective control groups (Fig. 3B). The % females laying eggs was lower (p < 0.05) when larvae and nymphs were treated in comparison to when adult stages were treated.

**Fig. 5:**
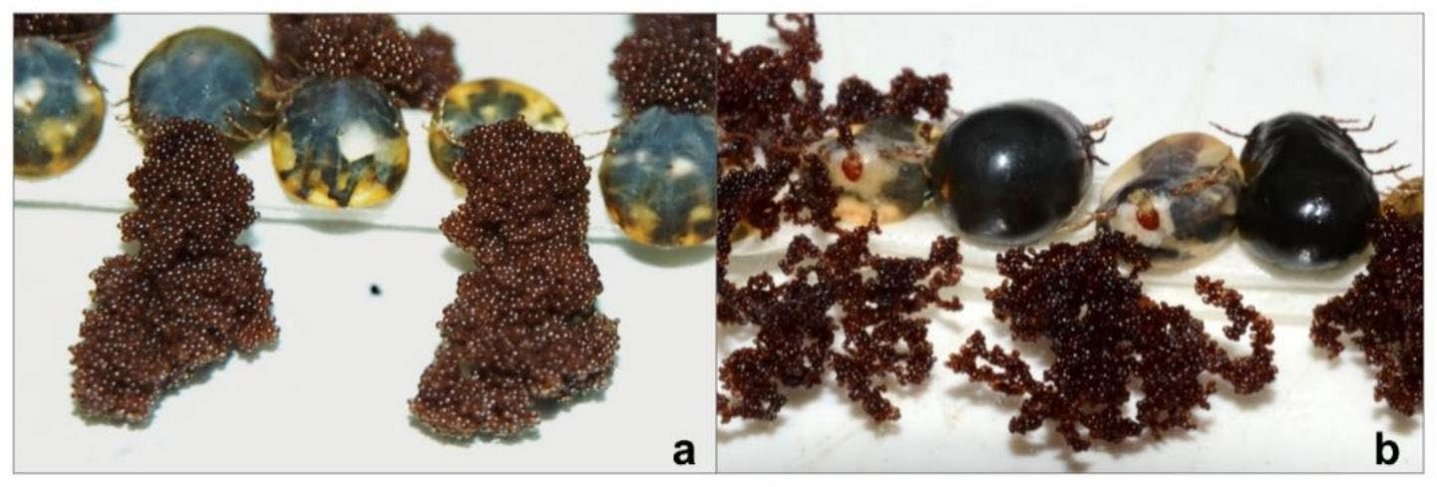
Effect of ethyl-4-bromophenyl carbamate on eggs produced by *Rhipicephalus microplus* engorged females in the pen test with infestation chambers. **(a)** Control ticks. **(b)** Engorged females sprayed on day 16 post-infestation

A group effect (F_5,117_ = 19.14, p < 0.0001) was observed on the % egg production. The % egg production was lower when nymphs (Tukey’s test: p < 0.01) and adults (p < 0.0001) were treated with ethyl-carbamate than their respective control groups (Fig. 3C). The treatment of adults with ethyl-carbamate resulted in lower egg production than when larvae (p < 0.0001) and nymphs (p < 0.01) were treated. In general, the eggs obtained from females treated at different stages were darker, opaque and dry, with deformed walls, and they easily disaggregated compared to eggs obtained from control females (Fig. 5).

A group effect (F_5,117_ = 24.99, p < 0.0001) was observed on the % egg hatching. The % egg hatching was lower when larvae (Tukey’s test: p < 0.05), nymphs, and adults (p < 0.0001) were treated with ethyl-carbamate compared to their respective control groups (Fig. 3D). The treatment of adults with ethyl-carbamate produced a lower % egg hatching than when larvae were treated (p < 0.02).

The cumulative reduction in larvae treated with ethyl-carbamate was 98.3%, and in treated nymphs and adults it was 96.1 and 94.4%, respectively (Table 1). The average cumulative reduction of hatched larvae, based on all three treated stages, was 96.3%.

**Table 1:** Mean (± SE) reduction (%) produced by ethyl-4-bromophenyl carbamate on various stages of *Rhipicephalus microplus* in a pen test with infestation chambers

Treated stage	Relative frequency of				Cumulative reduction (%) of hatched larvae
	engorged females	egg laying	egg production	egg hatching	
Larva	0.07 ± 0.030	0.78 ± 0.105	0.80 ± 0.071	0.38 ± 0.057	98.3
Nymph	0.28 ± 0.067	0.83 ± 0.039	0.71 ± 0.077	0.23 ± 0.052	96.1
Adult	0.73 ± 0.069	0.96 ± 0.011	0.45 ± 0.030	0.17 ± 0.041	94.4

## Discussion

Currently, the occurrence of strains resistant to conventional ixodicides on the market is the main limitation for the control of ticks in cattle, which urgently generates the development of new molecules effective against resistant tick strains. The development of a new ixodicide involves its design, *in silico* prediction, evaluation of its effects in vitro, and determination of its efficacy in vivo. The pen tests with infestation chambers were initially used to assess the efficacy of ixodicides on different stages of *R. microplus in vivo* (Downing et al. 1977). The results of this study showed that ethyl-4-bromophenyl-carbamate had a high efficacy on different stages of an *R. microplus* strain resistant to organophosphates, pyrethroids, and amidines (San Alfonso strain) implanted in cattle.

The efficacy of an ixodicide can be evaluated in various ways; one of them is to measure the efficacy of a treatment on parasitic stages based on tick survival, and another is to measure the efficacy based on egg production and egg hatching (Holdsworth et al. 2006). In the current study, we evaluated survival by measuring the developmental inhibition of engorged females. The spraying of *R. microplus* larvae implanted in bovines inhibited 93% of their development to engorged females because most of the larvae died, which shows the ixodicidal effect of ethyl-4-bromophenyl carbamate on larval stages. The efficacy of this ethyl-carbamate decreased when nymphs (72%) or adults (27%) were sprayed. This may be the result of apoptosis induced by this ethyl-carbamate on intestinal and salivary gland cells, as demonstrated by Escobar-Chavarría et al. (2021). These cells are likely more important for the survival of larvae and nymphs than for adults.

Efficacy based on egg production was evaluated through the % females laying eggs and the % egg production. The application of the carbamate in all stages of *R. microplus* was effective in reducing the number of eggs produced. This reduction is probably the result of the effect of ethyl-carbamate on reproductive tract cells, as observed by Prado-Ochoa et al. (2014c): when *R. microplus* females were treated with ethyl-carbamate in an adult immersion test (AIT), the development of the ovaries was inhibited, and the maturation of oocytes was affected. Iturbe-Requena et al. (2020b) observed processes suggestive of apoptosis by electron microscopy in the ovaries from females treated with AIT, and Escobar-Chavarría et al. (2021), using TUNEL-peroxidase and TUNEL-fluorescence assays, observed that this ethyl-carbamate induced apoptotic processes in cells from primary cultures of *R. microplus* ovaries.

The spraying of ethyl-carbamate on the three stages of *R. microplus*, not only decreased the number of eggs produced, but also decreased the number of eggs hatched. This decrease was greater when implanted adults were treated, probably because ovarian development, oogenesis, and oviposition occur at this stage, which compromised the viability of the eggs. In general, it was observed that the eggs obtained from females treated at different stages presented similar changes to those observed by Prado-Ochoa et al. (2013)d rez-González et al. (2014). Iturbe-Requena et al. (2020b), using DAPI fluorescent staining, observed inhibition of embryogenesis within eggs obtained from females treated in the laboratory with ethyl-4-bromophenyl carbamate. The results of this study showed that most of the eggs produced by treated females did not hatch, which could be related to the inhibition of embryonic development produced by the treatment of ticks implanted on cattle with ethyl-4-bromophenyl carbamate. However, the effect of this ethyl-carbamate on males that live on cattle and whether they play a role in the fertilization of females and in the embryogenesis of larvae inside the eggs is not known.

Reproductive potential is the ability of a population to produce viable offspring. We estimated how treatment with ethyl-4-bromophenyl carbamate affects this potential in *R. microplus*, calculating the percentage of cumulative reduction of larvae produced. The ethyl-carbamate decreased, on average, 96% of larvae produced for the next generation, which drastically reduced the number of individuals to establish a new cycle of infection. Furthermore, although it was not evaluated in this study, we do not know whether the larvae produced have the same behavior and ability to move in the pastures and infest other cattle.

In conclusion, ethyl-4-bromophenyl carbamate acted as an ixodicide (lethal effect) when larval stages of an *R. microplus* strain resistant to most conventional ixodicides were sprayed, and as a growth regulator when sprayed on nymphal and adult stages. The sum of these effects had a direct impact on the efficacy of the product in pen tests, and future studies will indicate the possible use of this product to control ticks in the field.

## Data Availability

All data and materials reported in this publication are available upon request.

## References

[CR1] Abbas RZ, Zaman MA, Colwell DD, Gilleard J, Iqbal Z (2014). Acaricide resistance in cattle ticks and approaches to its management: the state of play. Vet Parasitol.

[CR2] Alba-Hurtado F, Muñoz-Guzmán MA, Kumar (2021). Strategies for the control of *Rhipicephalus microplus* (Focus only on chemical acaricides). The Entomological Guide to *Rhipicephalus*.

[CR3] Andreotti R, Garcia MV, Higa LDOS, Piña FTB, Kumar (2021). Biology, ecology and importance of *Rhipicephalus* ticks in Brazil. The Entomological Guide to *Rhipicephalus*.

[CR4] Angeles E, Martínez P, Keller J, Martínez R, Rubio M, Ramírez G, Castillo R, López-Castañares R, Jiménez E (2000). Ethyl and methylphenylcarbamates as antihelmintic agents: theoretical study for predicting their biological activity by PM3. J Mol Struct THEOCHEM.

[CR5] Domingos A, Antunes S, Borges L, do Rosário VE (2013). Approaches towards tick and tick-borne diseases control. Rev Soc Bras Med Trop.

[CR6] Downing FS, Stubbs VK, Bowyer S (1977) Technique for localizing infestations of the cattle tick *Boophilus microplus* (Can.) on small areas of the host and subjecting each area to dip treatments. Crop Protection Agents Their Biological Evaluation. Proc Int Conf Eval Biol Act 609–622. https://agris.fao.org/agris-search/search.do?recordID=US201301273310

[CR7] Escobar-Chavarría O, Cossío-Bayúgar R, Ramírez-Noguera P, Prado-Ochoa MG, Velázquez-Sánchez AM, Muñoz-Guzmán MA, Angeles E, Alba-Hurtado F (2021). In vivo and in vitro apoptosis induced by new acaricidal ethyl-carbamates in Rhipicephalus microplus. Ticks Tick Borne Dis.

[CR8] George JE, Pound JM, Davey RB (2004). Chemical control of ticks on cattle and the resistance of these parasites to acaricides. Parasitology.

[CR9] Guerrero FD, Lovis L, Martins JR (2012). Acaricide resistance mechanisms in *Rhipicephalus (Boophilus) microplus*. Rev Bras Parasitol Vet.

[CR10] Holdsworth PA, Kemp D, Green P, Peter RJ, de Bruin C, Jonsson NN, Letonja T, Rehbein S, Vercruysse J (2006). World Association for the Advancement of Veterinary Parasitology (W.A.A.V.P.) guidelines for evaluating the efficacy of acaricides against ticks (Ixodidae) on ruminants. Vet Parasitol.

[CR12] Iturbe-Requena SL, Prado-Ochoa MG, Muñoz-Guzmán MA, Velázquez-Sánchez AM, Ángeles E, Alba-Hurtado F (2019). Toxic effects of new ethyl-carbamates on the morphology, mortality and acetylcholinesterase activity of *Eisenia foetida*. Ecotoxicol Environ Saf.

[CR11] Iturbe-Requena SL, Prado-Ochoa MG, Muñoz-Guzmán MA, Carrillo-Miranda L, Velázquez-Sánchez AM, Ángeles E, Alba-Hurtado F (2020). Acute oral and contact toxicity of new ethyl-carbamates on the mortality and acetylcholinesterase activity of honeybee (*Apis mellifera*). Chemosphere.

[CR13] Iturbe-Requena SL, Prado-Ochoa MG, Velázquez-Sánchez AM, García-Hernández F, Cossío-Bayúgar R, Muñoz-Guzmán MA, Ángeles E, Alba-Hurtado F (2020). Oogenesis and embryogenesis inhibition induced by two new ethyl-carbamates in the cattle tick *Rhipicephalus microplus*. Ticks Tick Borne Dis.

[CR14] NOM-016-ZOO- (1993) Norma Oficial Mexicana: Requisitos de efectividad biológica para los ixodicidas de uso en bovinos y metodo de prueba. https://www.Gob.Mx/Senasica/Documentos/Nom-006-Zoo-1993. Accesed 8 August 2022.

[CR16] Percie du Sert N, Hurst V, Ahluwalia A, Alam S, Avey MT, Baker M (2020). The ARRIVE guidelines 2.0: updated guidelines for reporting animal research. J Cereb Blood Flow Metab.

[CR15] Pérez-González IE, Prado-Ochoa MG, Muñoz-Guzmán MA, Vázquez-Valadez VH, Velázquez-Sánchez AM, Avila-Suárez BL, Cuenca-Verde C, Angeles E, Alba-Hurtado F (2014). Effect of new ethyl and methyl carbamates on *Rhipicephalus microplus* larvae and adult ticks resistant to conventional ixodicides. Vet Parasitol.

[CR21] Prado-Ochoa MG, Muñoz-Guzmán MA, Abrego-Reyes VH, Velázquez-Sánchez AM, Lara-Rocha M, Cuenca-Verde C, Angeles E, Alba-Hurtado F (2013). Effect of new ethyl and methyl carbamates on biological parameters and reproduction of the cattle tick *Rhipicephalus microplus*. Vet Parasitol.

[CR17] Prado-Ochoa MG, Abrego-Reyes VH, Velázquez-Sánchez AM, Muñoz-Guzmán MA, Ramírez-Noguera P, Angeles E, Alba-Hurtado F (2014). Subchronic toxicity study in rats of two new ethyl-carbamates with ixodicidal activity. Biomed Res Int.

[CR18] Prado-Ochoa MG, Gutiérrez-Amezquita RA, Abrego-Reyes VH, Velázquez-Sánchez AM, Muñoz-Guzmán MA, Ramírez-Noguera P, Angeles E, Alba-Hurtado F (2014). Assessment of acute oral and dermal toxicity of 2 ethyl-carbamates with activity against *Rhipicephalus microplus* in rats. Biomed Res Int.

[CR20] Prado-Ochoa MG, Ramírez-Noguera P, Díaz-Torres R, Garrido-Fariña GI, Vázquez-Valadez VH, Velázquez-Sánchez AM, Muñoz-Guzmán MA, Angeles E, Alba-Hurtado F (2014). The action of two ethyl carbamates on acetylcholinesterase and reproductive organs of *Rhipicephalus microplus*. Vet Parasitol.

[CR19] Prado-Ochoa MG, Muñoz-Guzmán MA, Vázquez-Valadez VH, Velázquez-Sánchez AM, Salazar AM, Ramírez-Noguera P, Angeles E, Alba-Hurtado F (2016). Genotoxicity and cytotoxicity assessment of new ethyl-carbamates with ixodicidal activity. Mutat Res Genet Toxicol Environ Mutagen.

